# Exploring Perceived Utility of a Clinic‐Integrated HIV Prevention App for Men Who Have Sex With Men in Malaysia: A Qualitative Think‐Aloud Study

**DOI:** 10.1002/hsr2.72458

**Published:** 2026-05-04

**Authors:** Md. Safaet Hossain Sujan, Jeffrey A. Wickersham, Antoine Khati, Kamal Gautam, Kiran Paudel, Iskandar Azwa, Frederick L. Altice, Yee Kee Tan, Nursahira Sahiba Mohd Sabri, Michael M. Copenhaver, Roman Shrestha

**Affiliations:** ^1^ Department of Allied Health Sciences University of Connecticut Storrs CT USA; ^2^ Section of Infectious Diseases, Department of Medicine Yale School of Medicine New Haven Connecticut USA; ^3^ Centre of Excellence for Research in AIDS (CERiA), Faculty of Medicine University of Malaya Kuala Lumpur Malaysia; ^4^ Nepal Health Frontiers, Tokha‐5 Kathmandu Nepal; ^5^ Infectious Diseases Unit, Department of Medicine, Faculty of Medicine University of Malaya Kuala Lumpur Malaysia; ^6^ Department of Epidemiology of Microbial Diseases Yale School of Public Health New Haven Connecticut USA

**Keywords:** HIV prevention, HIV self‐testing, Malaysia, mHealth, pre‐exposure prophylaxis, think‐aloud method, utility testing

## Abstract

**Background and Aims:**

HIV remains a global health concern, disproportionately affecting men who have sex with men (MSM). Discrimination, stigma, and other barriers in healthcare settings are common issues for MSM in Malaysia, resulting in sub‐optimal HIV testing and linkage to HIV prevention services. Thus, we created a clinic‐integrated HIV prevention app called “JomPrEP” to scale up HIV prevention efforts among this vulnerable group. This study aimed to explore the perceived utility, appeal, and functionality of the JomPrEP app for MSM in Malaysia to improve access to HIV prevention services.

**Methods:**

We conducted one‐on‐one qualitative interviews with 10 MSM and 10 healthcare stakeholders (i.e., physicians and administrators) using the ‘think‐aloud’ (TA) protocol. A brief example was provided to ensure participants understood the TA process, and then they were asked to open the app's web version in their browsers and share their screens. The TA procedure included 10 problem‐solving tasks, followed by a semi‐structured interview. These interviews aimed to delve deeper into the users' feedback on the pre‐specified tasks on nine features of the app. The TA sessions were video‐recorded and then transcribed verbatim. Theme analysis was used to analyze qualitative data.

**Results:**

MSM and stakeholder participants expressed most of the app features (e.g., app signup page, ordering HIV self‐test [HIVST] kits, and pre‐exposure prophylaxis [PrEP] medications) as being important and user‐friendly. Incorporating other features, particularly MedManager (e.g., a feature that sends reminders to users and tracks their medications) and reward systems (i.e., incentives for specific actions such as ordering HIVST or reviewing app resources), were well‐received. Conversely, participants also suggested (i.e., introducing edit and update options for PrEP and HIVST kit orders and incorporating confirmation messages for clinic appointments) to refine the app further.

**Conclusion:**

JomPrEP was found to be a functional, appealing, and useful platform to scale up HIV prevention services among MSM in Malaysia. Further refinements and pilot testing are needed to assess its impact on HIV prevention efforts for MSM in Malaysia.

## 1 Introduction

Despite evidence‐based treatment and prevention, HIV incidence continues to increase in some settings in the absence of scalable HIV prevention strategies [1], disproportionately affecting men who have sex with men (MSM), including in Malaysia [2]. Societal stigma and discrimination discourage MSM from disclosing their sexual orientation to their healthcare providers and accessing prevention and treatment services [3]. Much of this is related to anticipated or enacted stigma and discrimination in healthcare settings [3], which results in suboptimal utilization of HIV testing and prevention services. To more effectively scale up HIV prevention, innovative strategies that meet the needs of MSM in Malaysia are urgently needed.

Same‐sex behavior is criminalized in both secular and Shariah law in Malaysia [4], creating a barrier to engaging with HIV prevention efforts in person. An alternative strategy for MSM to interact with HIV prevention efforts is virtually using mobile health (mHealth), which can provide a range of behavioral interventions, guiding users to make better decisions and preventing diseases [5]. It also represents a significant advancement in HIV prevention strategies when integrated with existing evidence‐based interventions, such as HIV testing and pre‐exposure prophylaxis (PrEP) [6, 7, 8, 9]. For example, app‐based HIV prevention interventions can assist individuals in gaining knowledge of safe sex practices, monitoring sexual health, corresponding with clinicians, initiating PrEP, ordering sexual health products (i.e., HIV self‐testing kits, PrEP, condoms), and tracking medication [10, 11, 12, 13, 14].

In Malaysia, nearly all MSM own a smartphone and prefer to interface with “apps” instead of face‐to‐face interaction with a clinician to access HIV prevention and other support services (e.g., substance use, mental health) [15, 16]. An app‐based HIV prevention intervention can be a transformative approach to scale up HIV prevention among this marginalized and highly stigmatized population. Unfortunately, no such platforms are currently available for them in lower and middle‐income countries (LMICs) like Malaysia, even though these key populations are early adopters of mHealth technology [17, 18]. Thus, an ideal opportunity exists there to create an app for this stigmatized group to scale up HIV prevention services.

We created an interactive prototype of a clinic‐integrated smartphone app called “JomPrEP” for HIV prevention, aiming to increase HIV testing and PrEP uptake among MSM [14, 19]. Creating digital interventions, such as JomPrEP, necessitates an iterative process of revision and refinement [20]. Utility testing is a crucial step that allows the assessment of the perspectives of potential users and their acceptability [21]. Thinking‐aloud (TA) testing is an effective technique for assessing the utility of health behavior interventions [22, 23]. In this study, we, therefore, aimed to evaluate the JomPrEP app to gather feedback regarding its functionality, appearance, and perceived utility.

## 2 Methods

### 2.1 Study Design and Setting

The study employed one‐on‐one TA qualitative interviews with MSM and healthcare stakeholders (e.g., physicians, administrators, and outreach healthcare workers) from the Greater Kuala Lumpur region between February 2022 and April 2022. TA testing helps to refine the app by gathering participants' feedback regarding its appeal, perceived utility, and functionality [23].

### 2.2 Study Participants

The TA qualitative interviews were conducted with 20 participants, involving 10 MSM and 10 stakeholders. Participants were recruited through a convenience sampling technique. MSM participants were recruited by posting flyers on social media (e.g., Facebook, Instagram, Grindr) and through referrals from MSM‐serving organizations. The inclusion criteria for MSM were: (i) 18 years of age or above; (ii) self‐identify as cisgender MSM; (iii) self‐reported HIV negative or HIV status unknown; (iv) able to speak and understand Bahasa Malaysia or English; and (v) currently living in Malaysia. Inclusion criteria for healthcare stakeholders were: (i) age 18 years or above; (ii) employed as medical practitioners, nursing professionals, pharmacists, mental health counselors, community outreach workers, or non‐governmental organization (NGO) staff involved in providing HIV‐related services to MSM; and iii) able to speak Bahasa Malaysia or English. The stakeholders were recruited from MSM‐serving organizations.

### 2.3 JomPrEP App Prototype

This clinic‐integrated app was created based on our formative research as an additional platform to offer comprehensive HIV prevention services virtually [14, 19]. The app has been the same in both the previous and current study. By clinic‐integrating, we meant incorporating clinical services into the app, which allows participants to discreetly order HIVST kits, PrEP, and harm reduction tools (e.g., condoms) at their doorstep. In addition, the users can correspond with clinicians for e‐consultation, laboratory tests, and PrEP initiation. Table 3 gives a general overview of JomPrEP's key features, and Appendix 1 shows the app prototype.

### 2.4 Study Procedures

Interested participants who clicked flyers with a URL or scanned a QR code were directed to a self‐screening eligibility tool in Qualtrics. Subsequently, eligible individuals were contacted to assess their willingness to join the study and to invite them to participate in TA interviews.

Before the TA session, participants completed a brief anonymous survey that included MSM participants age, ethnicity, sexual orientation, engagement in anal and condomless sex, their current or past 6 months' HIV test results, ever using HIVST, ever or current usage of PrEP, and smartphone use. Likewise, stakeholders were asked about their age, ethnicity, profession, and professional longevity, and engagement in HIV and PrEP services.

The TA qualitative interviews were conducted using a video‐conferencing platform. Each session lasted approximately 90 min. The research staff assisted participants in accessing the web version of the JomPrEP app. The facilitator ensured participants understood the TA process by giving a brief example. After the tutorial, participants were asked to open the app in their browser and share their screens. The research staff then asked participants to complete 10 pre‐specified tasks (e.g., see Appendix 2) on features of the JomPrEP app. Participants provided feedback on the app's nine core features: app signup page, PrEP eligibility, and risk assessment using the smartsex checkup feature, appointment management system, HIVST kit, PrEP consultation and order, checking order status, MedManager, mental health screening and referral, rewards system, and providing their thoughts on the app interface.

Participants were advised to focus on the app's appearance, relevance, utility, and functionality during the TA session. During the task performance, participants were asked to continually verbalize their thoughts and activities, including visible aspects, emerging difficulties, and enjoyable elements. The participants actively interacted with the app, providing feedback on its functionality, navigation, and perceived utility as they expressed their thoughts and observations. The interviewer only intervened if the participants stopped thinking aloud and asked, “*What are your thoughts on this stage?*”

Following the TA test, participants were asked to complete a questionnaire on two main areas. They were also asked to evaluate the ease of completing the 10 pre‐specified tasks during the alpha testing session, using a rating scale from 1 (poor) to 5 (excellent). A trained facilitator, assisted by a co‐facilitator, led TA interviews with participants who could keep their cameras off and use pseudonyms. All sessions were conducted in Malay, English, or a mix, as per participants' preference, and were recorded for analysis. Participants were compensated RM40 (~US$10) for their time.

### 2.5 Ethics Statement

The Helsinki Declaration's recommendations on human engagement and institutional research ethics were followed in conducting the study. The University of Connecticut and the University of Malaya's Ethical Review Boards approved this study protocol. Before beginning the TA interview, the participants were informed of the study's purpose and procedures. They were also given information about data confidentiality and anonymity, the freedom to withdraw from the study at any time, and the freedom to do so. All participants provided verbal informed consent.

### 2.6 Data Analysis

Verbatim transcription and translation were performed on each video recording of TA sessions. The transcripts from MSM and stakeholders were carefully reviewed, and their accuracy was double‐checked before the thematic analysis. Three coders (MSHS, AK & KP) read and re‐read each transcription to identify key ideas and recurring themes. The coders independently developed their codebooks, followed by three meetings to iteratively discuss and cross‐check common and emerging themes, and to consolidate them into a final codebook by consensus. The results section is presented by theme, with representative quotes from participants. SPSS version 25.0.0 was used to calculate descriptive statistics for variables collected via a Qualtrics survey.

## 3 Results

### 3.1 Participants Characteristics

MSM Participants (*N* = 10): The mean age of participants was 28.0 {standard deviation (SD) = 0.1} years. As shown in Table 1, most participants (80%) were Malay, reported to have engaged in anal sex in the past 6 months (70%), tested HIV negative in the past 6 months (90%), and had never used PrEP (70%).

**Table 1:** Socio‐demographic characteristics of MSM (*N* = 10).

Characteristics		Total (*N* = 10), *n*%
Age	Mean age (SD)	28 (5.1)
**Ethnicity**		
Malay		8 (80%)
Chinese		1 (10%)
Native Sarawakian		1 (10%)
**Sexual Orientation**		
Bisexual		4 (40%)
Gay/PLU		6 (60%)
**Engaged in anal sex (past 6 months)**		
Yes		7 (70%)
No		3 (30%)
**Condomless sex (past 6 months)**		
Yes		3 (30%)
No		7 (70%)
**HIV test (past 6 months)**		
Yes		9 (90%)
No		1 (10%)
**Current HIV status**		
Unknown/Not sure		1 (10%)
HIV negative		9 (90%)
** Ever used an HIV self‐testing kit**		
Yes		5 (50%)
No		5 (50%)
**Ever used PrEP**		
Yes		3 (30%)
No		7 (70%)
**Currently using PrEP (*n* = 3)**		
Yes		2 (66.7%)
No		1 (33.3%)
**Currently using a smartphone**		
iOS		6 (60%)
Android		4 (40%)

*Healthcare Stakeholders* (*N* = 10): The mean age was 34.7 years (SD = 14.0). Table 2 shows that half of them (50%) were outreach workers, with less than half (40%) having 5 years in the profession. All healthcare facilities (100%) affiliated with the healthcare stakeholders provided HIV testing services for at least 5 years (50%). Most stakeholders (90%) were directly involved with HIV testing services and provided PrEP services (100%).

**Table 2:** Socio‐demographic characteristics of Stakeholders (*N* = 10).

Characteristics		Total (*N* = 10), n%
**Age**	Mean (SD)	34.7 (14.0)
**Ethnicity**		
Malay		2 (20%)
Chinese		3 (30%)
Native Sarawakian		2 (20%)
Indian		2 (20%)
Mixed ethnic		1 (10%)
**Current profession**		
Medical doctor		3 (30%)
Administrator		2 (20%)
Outreach worker		5 (50%)
**Years in the profession**		
< 5 years		6 (60%)
> 5 years		4 (40%)
**Current facility offers HIV testing**		
Yes		10 (100%)
No		0 (0%)
**Duration (in years) of your facilities providing HIV testing services**		
< 5 years		5 (50%)
> 5 years		4 (40%)
Can't remember		1(10%)
**Directly involved in HIV testing services**		
Yes		9 (90%)
No		1 (10%)
**Facility type**		
Clinic/Hospital‐private		3 (30%)
Clinic/Hospital‐governmental		2 (20%)
Community‐based organization		5 (50%)
**Current facility offers PrEP services**		
Yes		10 (100%)
No		0 (0%)
**Types of PrEP services**		
PrEP consultation/PrEP prescription		9 (42.9%)
PrEP dispensing (onsite pharmacy)		5 (23.3%)
PrEP delivery		4 (19%)
Online HIV and STI testing		2 (9.5%)
PrEP referral		1 (4.8%)
**Number of MSM who received PrEP‐related services**		
None		1 (10%)
1–10		5 (50%)
11–50		2 (20%)
50–100		2 (20%)
**Years of providing PrEP‐related services**		
< 1–5 years		8 (80%)
Can't remember		2 (20%)
**Directly involved in PrEP services (e.g, consultation, counseling, prescriptions)**		
Yes		8 (8%)
No		2 (20%)

*Note:* Variable name “types of PrEP services” recorded multiple responses.

### 3.2 Think‐Aloud Interview Results

Mixed feedback provided by MSM and stakeholders during the task performance is shown in Table 3.

**Table 3:** Summary of MSM and healthcare stakeholders' responses about JomPrEP app features (*N* = 10), *n* (%).

Features	MSM responses	Healthcare stakeholders' responses
**App signup page**	***N*, *n* (%)**	***N*, *n* (%)**
This is the landing page. Once the user downloads and opens the app, it leads them to a sign‐up page.	Column colors need to be brighter; the avatar is too small, the icons are blurry, passwords are less visible, and the terminology and birth date do not fit well with non‐English speakers; 7 (70%)	I think the color scheme could be more attractive, and they cannot see passwords while typing; 3 (30%)
	Auto‐refill information from Google or social media; limit the country code box up to 3 numbers and allow to customize the features like adding options to add photos; 3 (30%)	Add some soft, pastel colors to make the app more attractive; some people might prefer to register using a Google account or Facebook to make it easier; 6 (60%)
	User‐friendly and easy to navigate the registration process; 6 (60%)	It is a very straightforward and quick process that doesn't require too much personal information; 7 (70%)
**Order HIV self‐test kit (HIVST)**		
Users can order HIV self‐testing kits for themselves and a friend at their convenience and enter their shipping address to receive the order at their doorstep.	HIVST result uploading is a bit complicated, and many may not want to upload; the order section is separate from the HIVST kit section and is thus difficult to find, and users are redirected to the front page after the order is complete, which is confusing; 4 (40%)	Had some difficulty finding the correct area to click for the task and no clear indication of where and how you can upload your result; 2 (20%)
	To include more details after clicking on the HIVST, users need to see more details after they click on it, such as a shipping address, save the address for a future order, and provide images for the party pack; 5 (50%)	Use real names for packages rather than nicknames, and add contact information for someone who can help with questions; the video language could be regional with regional people; 5 (50%)
	Ordering an HIV self‐test kit was very easy; the interesting video with instructions made it more convenient and super‐fast; 6 (60%)	Easy, quick, and straightforward; 7 (70%)
**Smartsex Checkup**		
This is a risk‐behavior assessment tool to determine eligibility for PrEP.	Took some time to locate this feature, if miskicked, it cannot go back to the last question; 2 (20%)	Hard to find initially; some people don't know what STIs are but just recognize the names; 2 (20%)
	It would be better if shortened, added back and forward buttons, and more variety in graphics; 3 (30%)	Explain info about each STI for those who do not otherwise have access to that information; 1 (10%)
	It's simple, short, easy to read, and convenient for all age groups; 2 (20%)	Smart Sex checkup is a quick, comprehensive, and easy way to get information about HIV and STIs; 2 (20%)
**Appointment Management**		
The appointment feature enables users to schedule blood draws for laboratory tests and non‐judgmental online PrEP consultations. Users can easily book appointments for physical tests (i.e., chlamydia, gonorrhea, syphilis) and physician consultations at the selected clinics.	One can't reschedule the date, and there is no confirmation that your appointment date has been changed; 2 (20%)	No confirmation on the changed appointment date; no ‘date' appears to be able to be rescheduled; 2 (20%)
	Suggest informing the user that the clinic is closed so that they know why days are being skipped. Also, while rescheduling the appointment, need to mention the day so that the user doesn't get confused about which day the appointment has been rescheduled to; 3 (30%)	Add confirmation that your appointment has been rescheduled; it would be better if, after canceling the first appointment, we can reschedule it without doing the same process again; 3 (30%)
	Easy to see all the date and time choices and addresses listed in front of you so you can make your own choice; 3 (30%)	Very easy because all available appointment dates are listed, able to choose between pickup and delivery; 2 (20%)
**PrEP Express**		
This feature provides users with essential information about PrEP in an easy‐to‐access format. It offers detailed information about the different types of PrEP (same‐day PrEP, traditional PrEP, injectable PrEP, etc.) available and guidelines from health organizations to provide trusted and up‐to‐date information to help users make informed decisions about their sexual health.	Can't change the delivery options; insufficient information on the types of PrEP (traditional/rapid) to choose; better to provide the estimated time to receive the order based on the types of PrEP (traditional/rapid); 6 (60%)	Cannot order PrEP because it's just for negative people, and got a bit lost trying to find the area to order PrEP; 2 (20%)
	This feature should be in the order section with the time, prices, and discounts; need to integrate a feature that allows changing from delivery to pick‐up and vice versa; 6 (60%)	It would be better to mention somewhere else that PrEP is for negative people only so, positive people won't click; 4 (40%)
	Interesting app feature with precise and sequenced instructions for tracking order; 8 (80%)	In general, the information is enough; 1 (10%)
**Order History**		
The order history feature allows users to manage their orders for HIVST kits and PrEP medications efficiently. Users can monitor the status of their orders in real‐time, from processing and shipping to delivery. In addition, they can check the estimated delivery date and time, so they know the right time to expect their HIVST or PrEP medications. Furthermore, timely notifications are sent about order status, including shipment updates and delivery reminders, ensuring the users are always kept in the loop.	The font for “No current order“ is not bold enough, and did not notice that orders could be on that page; 3 (30%)	There is not much detail about the status; Need to repeatedly click on this same feature; 2 (20%)
	I suggest changing the icon to a lorry/truck or motor to indicate that the package has been delivered; give tracking details; 4 (40%)	Better to get a notification of the status of an order, and recommend placing a button in the HIVST kit ordering page to link participants/redirect them to the order status page to track the order; 3 (30%)
	The process is easy; 1 (10%)	There is not much detail about the status; need to repeatedly click on this same feature; 2 (20%)
**MedManager**		
This feature offers a comprehensive tool that helps users manage their PrEP and other medication schedules and improve adherence. It sends push notifications to remind users when it's time to take their PrEP or other medications. Users can also input their medication schedules, including remaining dosage and frequency, to keep an organized record of their medications.	Feels too wordy and not familiar with the medicine name; 3 (30%)	Not sure where the reminder message will come, confusion with “pills in stock“ twice, and can't move forward without adding the number of pills in stock; 3 (30%)
	Participants might not know the name of the medicine, so it would be better to have a drop‐down list of meds to choose from, remind users when they have a certain number of pills left, and add an easier button for clicking when they have taken a pill; 4 (40%)	Make the notification a “critical alert“ to override notification settings with more reminders and show the full month calendar with labels for days taken or skipped; 3 (30%)
	A very helpful feature for those who are on medication; the reminder message also has a lot of options, which is interesting and great; 2 (20%)	It is good for forgetful people, and keeping track of stock is easy and useful; 4 (40%)
**Mental Health**		
This feature helps users screen for depressive symptoms. The patient health questionnaire (PHQ‐9) is utilized in the app to assess their depression. If signs of moderate to severe depression are detected, the app provides a referral to consult with a healthcare professional. Participants can also contact for their mental health emergency through a given hotline number. They can also learn about depression with the accessible links given in this feature.	Most people have stress but no information on stress, and it takes a very long to read, and feel sleepy while reading; 3 (30%)	Screening for depression only undermines other disorders like anxiety, and heading for mental health is too vague; 3 (30%)
	Elaborate more briefly on every type of mental health information. People will click on whatever they like to read and add a consultant number to reach out anytime; 4 (40%)	Instead of just focusing on depression, it would be better to include varieties of mental health issues like anxiety, sleep, and suicidal ideation; 3 (30%)
	Mental health is a serious issue in MSM, so it's a very needed one; the referral letter is great because it makes us feel that we are cared for, and it is very easy to download. It's also convenient. the process of getting it through email is easy; 4 (40%)	Information is enough; referral letters are available. Also, the list of clinics so everything is okay; 3 (30%)
**Rewards**		
This feature aims to engage users and motivate them to continue using the app by providing financial incentives. Users earn points for completing any task within the app, such as ordering and purchasing HIVST kits and PrEP, screening for depression, tracking their mood, or managing their medication. Participants can redeem their earned points for cash at any time during the study.	Got confused about whether I got money or it was just a point and didn't like that bank information is required to use; 2 (20%)	Wants to see an explanation of how points work; 4 (40%)
	Think that the points should be used to deduct from the cost of getting PrEP, and it would be useful if participants could use the points in some other way, like e‐wallet or gift voucher; 4 (40%)	The information regarding points should be given during registration and instead of only having the option to redeem cash points, offering interesting gifts through the app can also be useful (e.g., a bottle of PrEP); 2 (20%)
	Easy, enjoyable, and straightforward process; 4 (40%)	Easy, interesting, and straightforward; 3 (30%)

### 3.3 App Signup Page

The majority of MSM (80%) and stakeholders (90%) expressed favorable views of the app signup page (e.g., a straightforward process that requires minimal information and time).

New account creation was seamless, simple, and straightforward.

[MSM #3]

Account creation was a straightforward process that doesn't require too much personal information.

[Stakeholder #19]

Almost 70% of MSM and 30% of stakeholders, however, reported some challenges while creating their new JomPrEP accounts (e.g., poor visibility of the password field and the use of unfamiliar terminology and date formats).

Cannot see the password as it is being typed.

[Stakeholder #2]

Terminology and language are not for non‐English speakers.

[MSM # 3]

About 30% of MSM and 60% of stakeholder participants suggested allowing users to auto‐fill information from their Google or social media accounts, adding some soft, pastel colors to make the app more attractive, and customizing features, such as adding options to upload photos of the app user. Although participants suggested minor refinements, 50% of MSM appreciated the feature, rating the app 5 out of 5, while 60% of stakeholders rated it 4 out of 5 for being straightforward.

Suggested signing in via Google or Facebook, whereby the information auto‐populates, instead of entering all the information yourself, which is tedious. Also, it would be easier if the phone number box were combined with the country code.

[MSM #18]

Notes that the background is “scary white” and reminds people of the hospital.

[Stakeholder #5]

### 3.4 HIV Self‐Testing Kit Order

A total of 80% of MSM and 70% of stakeholders noted the feature as very important, functional, easy to use, and self‐explanatory.

Obvious step‐by‐step instructions and videos helped with ordering. Excited to think that we can get results in 20 min.

[MSM #2]

Quick, easy, and adequate information.

[Stakeholder #11]

In comparison, 40% of MSM and 20% of stakeholders struggled to order HIVST kits as the order section and HIVST section were on separate pages.

The order section is separate from the HIVST kit section and is thus difficult to find. Users are redirected to the front page after the order is complete, which is confusing. Also, I tried to click on the HIV self‐test but couldn't.

[MSM #18]

Had some difficulty finding the correct area to click for the task.

[Stakeholder #4]

Additionally, 30% of MSM and 10% of stakeholders showed difficulty understanding how to upload HIVST results.

No clear indication on where and how you can upload your results.

[Stakeholder #7]

It is very difficult to upload the result.

[MSM #14]

Moreover, 50% of stakeholders suggested ordering HIVST kits and party packs using real names rather than pseudonyms. They also recommended including support staff contact information to help users assist with any questions. To better accommodate users who may not understand English well, they advocated making the app's supportive video available in multiple languages, including but not limited to Bahasa Malaysia and Mandarin.

Wants a little clarity on the name of the person the package will be addressed to (real name vs. nickname entered earlier).

[Stakeholder #6]

The video language could be regional with regional people.

[Stakeholder # 8]

### 3.5 SmartSex Checkup

While some participants (30% of MSM and 30% of stakeholders) provided positive feedback, some had difficulty locating this feature in the app.

Questions are understandable, and the risk assessment process is much easier. Enough comprehensive for PrEP.

[MSM #12]

Smartsex checks are an easy way to know and assess HIV and other STIs diseases conditions quickly. And this is suitable for HIV and other STI prevention.

[Stakeholder #7]

Took some time to locate this feature.

[MSM #17]

Hard to find initially.

[Stakeholder #11]

A total of 30% of MSM and 20% of stakeholders recommended some modifications, such as shortening the number of questions and adding back/forward navigation options. Despite this, participants found the SmartSex checkup feature very useful, with 50% of MSM and 20% of stakeholders rating it excellent.

It would be better if we shortened the survey.

[MSM #14]

Explain info about each STI for those who do not otherwise have access to that information.

[Stakeholder #5]

### 3.6 Appointment Management

Participants appreciated the appointment feature for its ability to schedule blood draws and doctor consultations at their convenience. They reported that the scheduling process was very easy.

Easy to see all the date and time choices listed and can schedule at a convenient time.

[MSM #5]

About 20% of participants from each group, however, encountered difficulties, such as being unable to cancel or reschedule appointments and not receiving a confirmation message despite successful rescheduling.

Cannot reschedule the date. Need to make a new appointment. It's tough to cancel the previous appointment and make a new one.

[MSM #14]

No confirmation on the changed appointment date. It is very tedious (need to redo the process again).

[Stakeholder #7]

In addition, 30% of participants from each group suggested some enhancements to the feature, such as adding notifications for appointment confirmations and allowing appointment rescheduling.

I suggested informing the user that the clinic is closed, so that they know why days are being skipped. Also, while rescheduling the appointment, you need to mention the day so that the user doesn't get confused about which day the appointment has been rescheduled.

[MSM #17]

Wants a confirmation with rescheduled information.

[Stakeholder #20]

### 3.7 PrEP Express

Almost 80% of MSM and 50% of stakeholders expressed the significance of PrEP services in the app and appreciated the incorporation of various PrEP information with accessible links.

Important feature with adequate information. Moreover, having a link to FAQs is useful (information can be accessed easily by a click), and having it collapsible as well.

[Stakeholder #19]

Interesting app feature. Sufficient information and instructions about PrEP provided.

[MSM #3]

About 60% of MSM and 20% of stakeholders emphasized the need for users to be able to modify PrEP order processing (e.g., delivery method, PrEP type), underscoring the importance of user choice and autonomy.

If you choose the delivery option, you cannot change to pick‐up anymore (really stressed this point).

[MSM #17]

Need to integrate a feature that allows changing from delivery to pick‐up and vice versa.

[Stakeholder #20]

Moreover, while performing this task, 40% of MSM and 40% of stakeholders pointed out challenges for first‐time users in choosing between traditional PrEP (a type of PrEP that requires blood draw and doctor consultation before PrEP prescription) and rapid PrEP (also known as same‐day PrEP, participants can be prescribed PrEP as they wait for the laboratory test results to come) due to the lack of information in the app.

Explain the difference between rapid PrEP and traditional PrEP within this feature.

[Stakeholder #4]

Not enough information on the types of PrEP.

[MSM #14]

### 3.8 Order History

Most participants appreciated this feature, which allowed them to track their HIVST kits or PrEP order status, while some (30% of MSM and 20% of community stakeholders) reported difficulties in locating it in the app.

Easy process but very helpful.

[MSM #12]

Did not notice that orders could be on that page. Users are redirected to the page, but it's otherwise difficult to find. When the HIVST order is complete, the users are redirected to the order section, whereas they would think they would stay on the same page.

[MSM #17]

I recommend placing a button (an option to link two separate pages in the feature) on the HIVST kit ordering page to link participants and redirect them to the order status page to track the order. This will make it easier, rather than navigating many times.

[Stakeholder #19]

Participants suggested improving the user experience by incorporating visual cues, such as using pictures to represent orders or highlighting the feature title within the app.

For the package, you can put a picture in this section (picture of the test kit and the party pack).

[MSM #17]

Wants pictures of everything that goes inside the kit.

[Stakeholder #19]

### 3.9 MedManager

About 40% of participants in each group appreciated the inclusion of the MedManager feature in the app, recognizing its potential to help users adhere to their medication schedules.

Beneficial feature for those on medication. The reminder message also has many interesting and great options.

[MSM #17]

It is good for forgetful people, and keeping track of medication stock is useful.

[Stakeholder #4]

Conversely, 30% of participants from each group reported a minor issue with the feature, such as unfamiliarity with the different medication names listed and being unable to proceed without specifying the number of pills remaining in stock.

Participants might not know the name of the medicine, so it would be better to have a drop‐down list of meds to choose from. Overall, the process is a bit challenging, especially if it is a first experience with these drugs (not familiar with them).

[MSM #18]

I cannot save without adding several pills in stock. I found a bug where a reminder isn't saved if the frequency is once a week or less, and I cannot unclick “take” if mistakenly clicked.

[Stakeholder #6]

In addition, 40% of MSM and 30% of stakeholders suggested changes, such as putting 3D pictures for each medicine and incorporating reminders when the medicine stock may run out soon. Interestingly, 30% of participants from each group rated it as an excellent app feature.

Can put the 3D picture regardless of its type and add the red color in the button.

[MSM #14]

More reminders that are more discreet (without the words med or pill) before running out of stock. Should be customizable so users can choose what pops up for them.

[Stakeholder #10]

### 3.10 Mental Health

Almost 70% of MSM and 50% of stakeholders appreciated this added feature, as it allowed the user to self‐assess depressive symptoms, a significant but often overlooked issue. Participants also reported that screening for depressive symptoms was easy and quick. Some participants, however, showed a mixed reaction to the feature.

Information is excellent and detailed; questions were brief and easy to understand.

[MSM #1]

Instructions are enough; referral letters are readily available. Also, it has a list of clinics. Everything seems perfect.

[Stakeholder #8]

This feature received a perfect rating of 5 out of 5 from 90% of MSM participants and community stakeholders. Additionally, 70% of participants suggested a broader focus on mental health by incorporating features that address stress and anxiety rather than only depression.

Most people have stress but no information on stress.

[MSM #12]

Screening for depression only undermines other disorders like anxiety.

[Stakeholder #15]

### 3.11 Reward System

Nearly 40% of MSM and 60% of stakeholders reported enjoying this feature in the app and rated it excellent, indicating that it would help with participant engagement and retention.

Very easy to use and enjoyed the reward system.

[MSM #1]

The participant will find “redeeming points” an interesting activity. Instead of only having the option to redeem cash points, offering interesting gifts through the app can also be useful (e.g., a bottle of PrEP).

[Stakeholder #15]

About 20% of MSM and 40% of stakeholders, however, reported being confused by this feature and did not understand what could be done with earned rewards.

Got confused about whether I got money or it's just a point.

[MSM #12]

Want to see an explanation of how points work?

[Stakeholder #20]

## 4 Discussion

The present study offers insightful information about how users interacted with the JomPrEP app, emphasizing both its perceived benefits and areas for improvement. In addition, the results advance our understanding of how best to optimize mHealth interventions to improve accessibility and engagement in HIV prevention initiatives.

Overall, participants found the JomPrEP app very appealing, functional, and useful to scale up HIV testing and PrEP uptake among MSM. They also suggested refinements to the app to further enhance its functionality and usability.

Our study showed that self‐assessment tools for mental health and PrEP eligibility (e.g., Smartsex checkup) were highly relevant and valuable for assessing behaviors, although some MSM and stakeholder participants suggested modifications to the feature to improve navigation and speed assessment. Making navigation easier and keeping the assessment questions shorter can make users more engaged with the platform. Existing research has shown that depressive symptoms are highly prevalent in MSM but are often overlooked [24]. They are also less likely to disclose their engagement in risky sexual practices (e.g., chemsex, group sex, condomless sex) to healthcare practitioners due to societal stigma and discrimination. Thus, online self‐assessment tools, which are often not part of standard care, could be quite beneficial for MSM in evaluating such behaviors and helping them consider appropriate health services (e.g., PrEP, mental health counseling) [25]. In addition, it empowers individuals to self‐assess their behavior and make informed decisions about their sexual health [26].

The online appointment management system, particularly for consultation with clinicians or laboratory tests, was another salient feature reported by MSM and stakeholders. In Malaysia, where same‐sex behavior is criminalized, such a platform that enables MSM to access care virtually helps mitigate stigma and discrimination in healthcare settings [4]. In support of our findings, recent research showed that telehealth can boost HIV prevention services, and making online appointments for laboratory tests and physician consultations can dramatically increase PrEP uptake and HIV testing [27, 28]. Another study by *Patel* et al. also demonstrated the effectiveness of virtual HIV and PrEP programs targeting MSM [29]. However, we have received suggestions to refine the feature, as it was difficult for some participants, in particular MSM, to reschedule or cancel appointments.

Most participants found the order feature very helpful as it allowed users to order various HIV prevention products (e.g., HIVST kits, PrEP medication, and harm reduction tools) privately and have them delivered to their doorstep. While this feature is relevant and important in Malaysia, as it minimizes multilevel barriers to accessing HIV prevention services for MSM [30], some participants from both groups suggested improving the user experience by adding pictures of HIVST kits or highlighting the feature title. Aligning with our study, prior research also showed that MSM mostly preferred buying HIVST kits through mobile apps, which can increase HIV and other sexually transmitted infections (STIs) testing rates [31] and linkage to PrEP services [32]. Scalability, convenience, and the non‐stigmatizing quality of mobile apps are some of the unique benefits of mHealth [33, 34].

Although participants from both groups in the present study noted that the medication reminder (i.e., MedManager) feature was highly relevant, functional, and useful, as it helped them by sending PrEP and other medication reminders and informing them about the remaining doses, they also shared some minor issues with the features, including a list of unfamiliar medication names that need to be addressed, and suggested adding 3D pictures for each medication type to improve understanding and providing tailored reminders when the medication may run out. Persistence and adherence to PrEP medications are key to preventing HIV. Research shows that PrEP is highly effective in preventing HIV infection, but its efficacy is reliant on adherence [35]. A recent study reported that participants did not adhere to PrEP after a certain period, as they tended to forget, lack motivation, and have challenges with consistent medication schedules [36]. A longitudinal cohort study of PrEP adherence showed that an app‐based reminder system increased PrEP adherence significantly [37].

In the present study, participants reported that the rewards feature was very interesting to them, while some participants in each group expressed confusion about the limited information on how the feature and the points would work. User engagement and retention are vital for the continued use of mHealth apps. Unfortunately, fatigue and lack of interest cause many users to quit using these apps shortly [38]. Earlier research suggested incorporating a financial incentives system in mHealth apps to continue the use of the apps and optimize medication adherence [39]. A retrospective and a longitudinal study also showed that a rewards system helps increase app engagement, minimize retention rate, and boost the continued use of the app [40, 41].

### 4.1 Strengths and Limitations

The strength of this study is rooted in its incorporation of MSM and healthcare stakeholder perspectives to evaluate the app's appearance and perceived utility. In addition, we first designed and created an app for lower and middle‐income countries (LMICs) like MSM in Malaysia to receive HIV prevention benefits online, as discrimination and stigma are highly prevalent in in‐person healthcare visits [4, 42]. Moreover, this app may promote PrEP uptake and increase HIV testing, resulting in lowering HIV rates among this vulnerable group. The sample size utilized in our study may appear to be small, though prior studies have demonstrated that 80%‐90% of technological issues in apps or websites can be identified in a sample of 5 to 9 participants, and TA strategies use similar sample sizes [43, 44]. Furthermore, our app provides an anonymous clinical service (e.g., users are not obligated to provide personally identifiable information and can use discreet app icons to hide the app from others), ensuring users' privacy and confidentiality. Our study incorporated mostly younger participants, those most at risk for HIV. A study found that young adults, compared to older people who own smartphones, have a shorter learning curve, a higher rate of performance, a lower error rate, better long‐term retention, and greater subjective satisfaction [45]. A further strength of our study is that it employs the TA approach, which can record rapid emotions and thoughts as users navigate the app. The app's next version will include multiple changes based on input from this study. However, the refinements suggested during alpha testing, such as auto‐filling personal information from Google or social media, which might pose privacy and user safety risks, were approached with caution. Finally, we will conduct a study to assess the app's impact on HIV prevention among MSM in Malaysia.

Despite its important findings, this study has some limitations. First, participants utilized the app's web version rather than downloading it to their devices. This may have influenced their experience and the feedback on the app's functionality and performance. Second, the JomPrEP app may not be helpful for everyone since it was tailored specifically for MSM in Malaysia. Third, selection bias may have occurred because we may have recruited MSM who were more accustomed to using mobile apps than others (i.e., hidden MSM) by recruiting MSM who used social media. Fourth, the language of JomPrEP is solely English. Thus, linguistic obstacles may limit MSM who would otherwise be eligible to participate in this research. Finally, we did not specifically ask about stakeholders' sexual orientation, which could potentially lead to provider bias.

## 5 Conclusion

Participant feedback from the TA sessions highlighted satisfaction with the JomPrEP app's appearance, functionality, and utility, though minor adjustments were suggested to refine it. Based on feedback, we cautiously refined the app, proceeded to beta testing, and will conduct a randomized controlled trial study to determine its effectiveness in promoting HIV testing and PrEP uptake among MSM in Malaysia.

## Author Contributions

**Md Safaet Hossain Sujan:** writing in original draft, validation, methodology, formal analysis, data curation, visualization. **Jeffrey A Wickersham:** writing in review and editing, validation. **Antoine Khati:** validation, writing in review and editing. **Kamal Gautam:** validation, writing in review and editing. **Kiran Paudel:** validation, writing in review and editing. **Iskandar Azwa:** validation, writing in review and editing, resources, project administration. **Frederick L Altice:** writing in review and editing, validation. **Yee Kee Tan:** writing in review and editing, validation. **Nursahira Sahiba Mohd Sabri:** validation, writing in review and editing. **Michael M Copenhaver:** writing in review and editing, validation. **Roman Shrestha:** conceptualization, investigation, funding acquisition, writing in review and editing, validation, methodology, project administration, supervision, resources.

## Conflicts of Interest

The authors declare no conflicts of interest.

## Transparency Statement

The lead author Roman Shrestha affirms that this manuscript is an honest, accurate, and transparent account of the study being reported; that no important aspects of the study have been omitted; and that any discrepancies from the study as planned (and, if relevant, registered) have been explained.

## Data Availability

Data from this study may be obtained from the corresponding author upon reasonable request.

## Appendix 2 Alpha Testing Think Aloud Interview Guide

Participants were told to:

*Think aloud while performing your tasks and pretend the facilitator is not there. Do not turn to assistance from the facilitators. If you fall silent for a while, the facilitator will remind you to keep thinking aloud. Finally, remember that it is the smartphone app, not you, who is being tested.*

Participants were asked to complete the following tasks using the app wireframe:

- Task 1: Show us how to register on the app
- Task 2: Show us how to order an HIV self‐testing kit
- Task 3: Show us how to schedule an e‐consultation with the clinician
- Task 4: Show us how to get information about PrEP
- Task 5: Show us how to contact someone on the research team about research study‐related issues
- Task 6: Show us how to schedule an appointment for labs
- Task 7: Show us how to order PrEP medication
- Task 8: Show us how to log your medication intake
- Task 9: Show us how to complete the weekly check‐in
- Task 10: Show us how to track the shipment of your HIV self‐testing kit order

Following the testing, the participants were asked to complete a questionnaire that contained questions on two main aspects:

Participants' experiences on the testing

On a scale of 1 to 5, how easy was it to understand the facilitator leading the testing?

Participants' experience using the wireframe app to complete tasks.

On a scale of 1 to 5, how easy was it to complete:

▪ Task 1: Show us how to register on the app
▪ Task 2: Show us how to order an HIV self‐testing kit
▪ Task 3: Show us how to schedule an e‐consultation with the clinician
▪ Task 4: Show us how to get information about PrEP
▪ Task 5: Show us how to contact someone on the research team about research study‐related issues
▪ Task 6: Show us how to schedule an appointment for labs
▪ Task 7: Show us how to order PrEP medication
▪ Task 8: Show us how to log your medication intake
▪ Task 9: Show us how to complete the weekly check‐in
▪ Task 10: Show us how to track the shipment of your HIV self‐testing kit order

In addition, the questionnaire will have space for additional comments.
